# Birth Trends among Female Physicians in Taiwan: A Nationwide Survey from 1996 to 2013

**DOI:** 10.3390/ijerph14070746

**Published:** 2017-07-09

**Authors:** Yi-Jen Wang, Shu-Chiung Chiang, Tzeng-Ji Chen, Li-Fang Chou, Shinn-Jang Hwang, Jui-Yao Liu

**Affiliations:** 1 Department of Family Medicine, Taipei Veterans General Hospital, 201, Taipei 112, Taiwan; sunnyclairew@gmail.com (Y.-J.W.); tjchen@vghtpe.gov.tw (T.-J.C.); sjhwang@vghtpe.gov.tw (S.-J.H.); 2 Institute of Hospital and Health Care Administration, National Yang-Ming University, Taipei 112, Taiwan; scchiang0g@gmail.com; 3 Department of Financial Engineering and Actuarial Mathematics, Soochow University, Taipei 100, Taiwan; 4 School of Medicine, National Yang-Ming University, Taipei 112, Taiwan; 5 Department of Public Finance, National Chengchi University, Taipei 116, Taiwan; lifang@nccu.edu.tw

**Keywords:** birth trend, delayed childbearing, maternal age, reproductive behavior, female physicians

## Abstract

*Background*: Although more and more women are becoming physicians, their decisions regarding pregnancy may be affected by the lengthy period of medical education and postgraduate training. The aim of this study was to explore the birth trends among female physicians in Taiwan; *Methods:* Retrospective analyses of maternal ages at delivery from 1996 to 2013, both for physicians and the general population, were conducted using a nationwide dataset called National Health Insurance Research Database; *Results*: During the study period, 8540 female physicians were identified. The physicians delivered a total of 4940 births in that time, with a rise from 210 in 1996 to 440 in 2013. In addition, the mean maternal age of the physicians at delivery increased from 32.19 years (standard deviations (SD) 2.80) in 1996 to 33.61 (SD 3.21) in 2013, values significantly higher than those for non-physicians of 27.81 (SD 4.74) in 1996 (*p* < 0.001) and 31.36 (SD 4.78) in 2013 (*p* < 0.001); *Conclusion*: Female physicians usually gave birth at an older age than non-physicians, but the discrepancy between the two groups gradually declined over the 18-year course of the study. The establishment of a maternity-friendly environment for female physicians should be considered by those who determine healthcare system policies.

## 1. Introduction

The number of women entering the medical field has increased dramatically in recent years. In 1996, women accounted for just 17.9% of medical school graduates in Taiwan, but that rate had risen to 26.6% as of 2014, with similar increases in the levels of female physicians having also been noted in other countries around the world [1,2,3,4,5]. Meanwhile, female physicians may face specific concerns with respect to their reproductive health. Physicians are generally recognized as bearing heavy workloads, including long working hours and high levels of shift work, and facing greater job stress than most other professionals. Previous studies have shown several work-related adverse effects on pregnancy outcomes, such as increased risks of preterm delivery from working at night, from long working hours (e.g., more than 40 h per week), and from jobs that require standing for extended periods (e.g., more than 3 h per day), as well as lower birth weights due to working hours more than 40 h per week and an increased risk of miscarriage from working 71 h per week [6,7]. Struggles to overcome barriers in balancing work and gender roles remain a difficult issue for female physicians, especially in a society with stereotypical gender roles [8]. Furthermore, female physicians are often discriminated against if they take maternity leave, may be annoyed by interruptions during internships and residency training, and can be rendered more vulnerable than male physicians by the physical and mental risks of reproduction; therefore, they are more likely to postpone childbearing until after they have completed medical school and residency training [9,10,11,12]. An advanced maternal age, however, carries various reproductive risks, such as infertility, miscarriage, the necessity of cesarean delivery, preterm labor, low birth weight, and congenital anomalies [13,14]. Maternal health and neonatal outcomes are a topic of particular concern for surgeons, urologists, obstetricians, and gynecologists, as those specialties require particularly long periods of training, such that reproductive considerations may have an impact on women’s choices of specialty in their medical careers [9,10,11].

Taiwan, as a country with a very high level of human development, has faced a declining birth rate since the baby boom period from 1946 to 1964, a factor which has combined with increased longevity to yield a rapidly aging population. The total fertility rate in 2015 was 1.18 births per woman, far below the ideal replacement level of 2.1 needed to keep a population from shrinking, and places Taiwan on the list of countries with the lowest birth rates in the world [15,16]. A low birth rate is a challenging issue that can eventually lead to diminished economic growth and lower living standards, due to the resulting decrease in population [17]. In developed countries, the factors that discourage women from childbearing can include employment, pursuing higher education, lifestyle choices, easy access to contraceptives, infertility, the risks or fears associated with late childbearing, and socio-economic barriers including dysfunctional pregnancy/maternity leave policies, as well as high costs of living and childrearing [18,19]. Moreover, higher educational levels, higher incomes, less flexible work, delayed marriage, an increasing unmarried population, and advanced maternal age are all positively associated with lower fertility [20,21]. Across parts of Europe and East Asia, delayed childbearing among a great number of career-oriented women would even exacerbate the falling fertility rate [13,18]. Female physicians of fertile years, as a group, are highly educated and have relatively high incomes. The aim of this study was to explore the birth trends among female physicians in Taiwan in order to determine their childbirth characteristics, as compared with those of non-physicians.

## 2. Materials and Methods

### 2.1. Data Collection

Our nationwide data came from Taiwan’s National Health Insurance Research Database (NHIRD), a database that contains the registration files and originals claim data for reimbursements from Taiwan’s national health insurance (NHI) program; data sets from the NHIRD are provided to academic researchers upon request [22]. More specifically, we used data from the registry for medical personnel (PER) data files, a set of registration files included in the larger NHIRD that contains the medical information of registered health professionals, including the identification number, sex, birthday, and job category of each individual medical personnel. For the period from 1996 to 2013, the data set included information on 8735 female physicians. Among those female physicians, 24 for whom a birthdate was not identified were excluded from the data analysis. One hundred sixty-seven people born before 1940 were excluded under consideration of irrelevancy for childbearing during the studied period. Four people born after 1995 were also excluded under the rationale that they were not likely to have graduated yet from medical school at such young ages. Medical students in Taiwan are typically required to complete 7-year program leading to the degree of Doctor of Medicine, and they are qualified for the board certification exam afterwards. Finally, data for 8540 female physicians were ultimately included for further analysis.

In addition, we used the inpatient expenditures by admissions (DD) data files, which contain whole original claims data for inpatients, including identification number, birthdate, date of admission, and notations regarding copayment exemptions, where the code “002” indicates labor and delivery. For relevant cases, the maternal age at delivery was calculated by subtracting the birthdate from the date of admission. Women with same identification numbers being identified via the PER data files were marked as physicians, otherwise, as non-physicians. Those women in the overall data set who gave birth were divided into physician and non-physician groups. The year, birth number, and maternal age at delivery were recorded for the two groups. Primipara mothers, meaning women giving birth for the first time, and multipara mothers, meaning women with multiple deliveries, were not classified.

The government of Taiwan launched the single-payer NHI program in 1995, and this universal health care program currently covers more than 99% of Taiwan’s population of approximately 23-million [23]. The conduct of the study and its procedures were approved by the institutional review board (2013-04-005E) of Taipei Veterans General Hospital, Taipei, Taiwan.

### 2.2. Qualitative Analysis

A retrospective, observational cohort analysis was conducted using the nationwide data from 1996 to 2013. Descriptive and inferential statistics were produced. For the both the female physician group and the non-physician group, we compared the maternal age at delivery in 1996 and to the maternal age at delivery in 2013. In addition, we also compared the two groups with one another with respect to the maternal ages at delivery in 1996 and 2013. Independent t tests were conducted using a 95% confidence interval (CI). A *p* value < 0.05 was regarded as significant. The programming software Perl (version 5.20.1, Perl Foundation, Walnut, CA, USA) was used for data processing and computations. Descriptive statistics and graphs were displayed using the statistical programming software R (version 3.2.4, R Foundation for Statistical Computing, Vienna, Austria) and Excel 2013 (Microsoft Corporation, Redmond, WA, USA).

## 3. Results

Among the 8540 female physicians identified, the vast majority were born after the 1960s, with 19.8% having been born in the 1960s, 34.9% having been born in the 1970s, and 34.8% having been born in the 1980s.

A total of 4,067,963 labors and births were identified among the entire study cohort, with 4940 (0.12%) births among the physicians and 4,063,023 (99.88%) among the non-physicians during the 18-year study period. Overall, the number of births decreased with time, from 307,101 births in 1996 to 191,621 in 2013. The two major exceptions to the overall trend of decreasing births occurred in 2000, when the number of births saw a 6.8% increase, and 2012, when the birth rate increased by 18.0%. The maternal age at delivery and the number of births among physicians and non-physicians in each year are listed in detail (Table 1).

**Figure 1:**
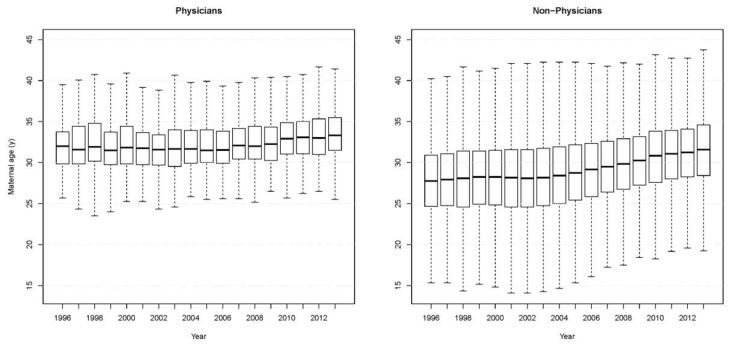
Distribution of maternal age at delivery among female physicians and non-physicians, 1996–2013.

The number of births among physicians doubled from 1996 to 2013. On average, female physicians gave birth at the age of 31 to 33 years, with standard deviations (SDs) ranging from 2.80 to 3.39 years. The physicians gave birth solely in adulthood (age 20 and over). A significant difference in the mean age at delivery of the physicians was found between 1996 (32.09 ± 2.80 years) and 2013 (33.61 ± 3.21 years) (*t* (465.8) = −6.16, 95% CI 2.01–1.04, *p* < 0.001). The box plots for the maternal age at delivery in each year are illustrated in Figure 1.

As to the non-physicians, the mean age at delivery was 27 to 31 years, with standard deviations ranging from 4.59 to 5.01 years during the study period. Some of the non-physicians gave birth in their teens (age under 20). A significant difference in the mean age at delivery was found between 1996 (27.81 ± 4.74 years) and 2013 (31.36 ± 4.78 years) (*t* (402470) = −255.17, 95% CI 3.57–3.52, *p* < 0.001).

The average age at delivery increased with time in both groups, but the maternal ages at delivery of the physicians were two to four years higher than those of the non-physicians in all the years of the study period. The mean age at delivery of the physicians was significantly older than that of the non-physicians in 1996 (*t* (209.8) = 22.08, 95% CI 3.90–4.66, *p* < 0.001). A similar statistically significant finding was also noted in 2013 (*t* (443.5) = 14.70, 95% CI 1.96–2.56, *p* < 0.001). On the whole, the female physicians had narrower ranges of maternal ages at delivery over the study period (Figure 1).

## 4. Discussion

### 4.1. Main Findings and Possible Explanations

The results of this study showed a sharp increase in the number of women physicians paired with a rising number of births among female physicians during the study period. Possible explanations for this increasing trend include more stable job statuses and incomes, protective work-related policies such as pregnancy/maternity leaves, manageable working hours, and accommodations for breastfeeding, as well as comprehensive access to reproductive health knowledge and resources. However, none of the female physicians gave birth before the age of 20, a finding which could lead one to infer that teen pregnancy and birth make medical education and careers difficult to achieve, in addition to being something considered blameworthy due to social and cultural norms. Therefore, the general fertility rates, which derive from the average live births to 1000 childbearing-aged women (aged 15–49) in a given year, could not be counted for female physicians. In contrast, some of the non-physicians gave birth in the beginning of their reproductive years, say, from 15 to 19 years of age. Teenage pregnancy and birth often raise the concerns of higher risks of single parenthood, economic deprivation, being dependent on social assistance, interrupted schooling, and difficulties entering the labor market; various studies, in fact, have found an evident association between deprived socioeconomic status and teenage pregnancy [24,25,26]. Meanwhile, the maternal age at delivery of the elderly maternal population in both groups was around 40 years of age. For women over 40 years of age or experiencing menopause, the success rates in terms of live births and even achieving pregnancy, whether by spontaneous conception or assisted reproduction, decrease substantially in comparison to younger women [13].

In the present study, the average ages of childbirth for the female physicians were 31 years and over, higher than those of the non-physicians in all the given years. Such advanced maternal ages at delivery among the female physicians was similar to the findings from the Unites States that the average age at delivery was 29.29 years for residents training in family medicine, and 32.6 years for urologist to have the first child [9,27]. Several factors that may deter female physicians from childbearing have been posited, such as the stigma associated with pregnancy during an internship or residency training, perceived career threats, a lack of support from colleagues, inconsistent maternity/parental leave policies, and inflexible training programs undertaken to meet board or specialty certification requirements [8,10,12,28]. The ideal time and the time at which a substantial proportion of female physicians, especially surgeons, choose to become pregnant is after the completion of their training, such as when they are in independent practice [10]. Faced with the rapid increase in the overall number of female physicians, most healthcare institutions have lagged behind in building up sound policies and secure environments with regard to maternity leaves and breastfeeding. Although the Labor Standards Act implemented by Taiwan’s Ministry of Labor included regulations on maternity leaves, physicians in Taiwan, including female physicians, are among the vulnerable groups currently not protected under any labor law. In 2011, the Ministry of Health and Welfare announced suggested regulations regarding workload adjustments during pregnancy, on-duty hours (including an exemption from night work between 10 p.m. and 6 a.m.) during pregnancy or breastfeeding, and breastfeeding sessions for female physicians. However, further improvements in constructing maternity-friendly working environments could be made regarding paid maternity leave and paid paternity leave. Furthermore, policies aimed at providing institutional support for pregnancy, parenting, and babysitting, may have positive effects on future birth trends among female physicians. Despite the fact that their collective mean age at delivery increased 1.33 years during the 18-year study period, the female physicians nonetheless varied less in their ages at delivery than did the non-physicians. As a group, the mean age at delivery of the non-physicians substantially increased over the entire study period, rising by 3.55 years overall and exceeding 30 years of age in 2009, with further increases in the following four years. Delayed childbearing is known to be associated with adverse obstetrical and perinatal outcomes, as well as increased risks to maternal health, such as an increased risk of breast cancer [13,14,29]. The Society of Obstetricians and Gynaecologists of Canada has recommended that women not delay childbearing beyond 32 years of age due to the significant declines in fecundity and fertility that most women experience beyond that age [30]. As such, the delays that women have already been making deserve detailed explorations on the parts of policymakers and scholars. On the other hand, with regard to early childhood development, studies conducted in the United Kingdom and Japan have posited that higher maternal ages are beneficial in terms of children experiencing fewer unintentional injuries, fewer hospital admissions, increased immunization rates, better language development, and less social and emotional difficulties [31,32]. However, whether similar findings would be found among the population in Taiwan should be examined further. Nonetheless, institutional policies should be enacted to protect the susceptible population, that is, potential mothers of all ages.

In this study, the annual number of births in the general population declined rapidly over the study period, decreasing by 37.6% from 1996 to 2013. This result reflected the fallen birth rate being calculated by dividing the number of the women of reproductive age (15–49 years) by the number of births in one year, with a decrease from 54 live births to 1000 childbearing-aged women in 1996, to 32 in 2013 [16]. This finding was similar to findings for many Organisation for Economic Co-operation and Development (OECD) countries such as the United States, United Kingdom, Germany, Korea, and Japan [24]. Despite transient surges in the numbers of births in 2000 and 2012, both of which were years of the dragon in the Chinese calendar, and thus years in which families were incentivized to give birth due to the auspicious qualities of said years; this ‘dragon baby boom’ phenomenon had little impact on the falling birth rate in the long run. As such, Taiwan faces not only a shrinking workforce, but a rapidly aging population. The declining birth rate will thus necessitate the evolution of related policies. The government has recently tried several policies to tackle the flagging birth rate, including providing subsidies for child care and fertility treatments, and offering cash incentives for each birth. However, Taiwan’s policymakers should go further to construct supportive institutional systems to offset the pressures derived from demographic changes.

### 4.2. Study Limitations and Strengths

There were limitations to this study. The retrospective sample data did not include data regarding some variables that would affect childbearing, such as marital statuses, so potential confounding factors could not be controlled. Also, data on births in foreign countries or deliveries which were not covered by the NHI program were not included in the database. Data regarding certain demographic factors, such as individuals’ socioeconomic situations, which may affect women’s choices regarding pregnancy, were not considered in the study under the rationale that a substantial amount of women discontinue employment after marriage or pregnancy [33]. We could not identify their professional occupations other than medical personnel, either. In addition, data regarding birth parity, complicated pregnancies, labor and delivery complications, and maternal health statuses were not considered in the present study. The rates of pregnancy induction, cesarean delivery, and the use of assisted reproduction technology were also not included. The relationship between the birth rate and certain demographic factors or methods of delivery should thus be controlled for and evaluated further in future studies. Furthermore, data regarding the working statuses, duty hours, workloads, rates of night shift work, maternal leaves, medical hierarchies of physicians, and differences across medical fields among the female physicians were likewise not known. In well-developed countries with robust social support systems like Denmark and Finland, the influence of psychosocial job strain on obstetric risks appears to be small, with the obstetric outcomes among female physicians and women of similar socio-economic backgrounds being similar [34,35]. Further investigations regarding the impacts of modifiable work-related and institutional factors on pregnancy planning and success in childbirth should be carried out.

Despite these limitations, our study demonstrates the maternal age at delivery and number of births among the female physicians in comparison with the non-physicians analyzing eighteen years of data on a population level. By observing the discrepancies between the birth trends among the female physicians and the non-physicians, we could indirectly understand childbearing and related reproductive behaviors in these two distinct groups. The information derived from our study should be of interest to those responsible for promoting women in medical careers and would contribute to possible policy implementation against delayed childbearing and falling birth rate.

## 5. Conclusions

In this study, declining numbers of births and an increasing maternal age at delivery were noted for the general population in Taiwan. This demographic profile change brings the prospect of a shrinking workforce, as well as concerns about increasing care burdens placed on the working-age population due to an increase in the aged population. However, the study results regarding female physicians in particular indicated that they had children later in life, and delayed childbirth to a lesser degree over time, than did the non-physicians. These findings have important implications for future research and policy implementation, including the identification of institutional and socio-economic factors that may impact childbirth and the construction of maternity-friendly environments for female physicians.

## Figures and Tables

**Table 1:** Maternal age at delivery and number of births among female physicians and non-physicians, 1996–2013.

Year	Physicians			Non-Physicians		
	Births (N)	Maternal Age (years)		Births (N)	Maternal Age (years)	
		Median	Mean ± SD		Median	Mean ± SD
1996	210	32.00	32.09 ± 2.80	306,891	27.75	27.81 ± 4.74
1997	193	31.58	31.97 ± 3.16	304,722	27.92	27.97 ± 4.73
1998	200	31.92	32.45 ± 3.22	249,546	28.08	28.09 ± 4.94
1999	244	31.50	31.94 ± 3.23	260,704	28.25	28.23 ± 4.82
2000	214	31.83	32.22 ± 3.20	278,368	28.25	28.29 ± 4.87
2001	203	31.75	31.84 ± 2.95	240,890	28.17	28.18 ± 5.01
2002	219	31.58	31.85 ± 3.08	233,401	28.08	28.19 ± 4.99
2003	236	31.67	32.02 ± 3.39	219,594	28.17	28.32 ± 4.97
2004	221	31.67	32.13 ± 3.10	212,088	28.42	28.53 ± 4.93
2005	241	31.50	32.04 ± 3.16	202,114	28.75	28.85 ± 4.92
2006	254	31.54	32.15 ± 3.16	200,042	29.17	29.15 ± 4.82
2007	291	32.08	32.41 ± 2.95	198,331	29.50	29.52 ± 4.71
2008	305	32.00	32.62 ± 3.39	191,933	29.83	29.85 ± 4.69
2009	318	32.25	32.45 ± 3.13	188,249	30.25	30.22 ± 4.61
2010	328	32.92	33.05 ± 2.92	162,392	30.83	30.64 ± 4.76
2011	391	33.08	33.32 ± 3.07	193,829	31.08	30.89 ± 4.61
2012	432	33.00	33.32 ± 3.29	228,748	31.25	31.07 ± 4.59
2013	440	33.33	33.61 ± 3.21	191,181	31.58	31.36 ± 4.78

SD: standard deviation.

## References

[1] Taiwan Medical Association (2015). Statistics yearbook of practicing physicians and health care organizations in Taiwan. http://www.tma.tw/tma_stats_2015/2015_stats.pdf.

[2] Huang H.L., Lee K.Y. (2003). The exploratory research on distribution of female physician manpower in Taiwan. Taiwan Med. J..

[3] Association of American Medical Colleges (2014). 2014 Physician Specialty Data Book.

[4] British Medical Association 2014 UK medical workforce briefing. https://www.bma.org.uk/-/media/files/pdfs/working%20for%20change/policy%20and%20lobbying/uk%20medical%20workforce%20briefing%20may%202015%20final.pdf.

[5] Ramakrishnan A., Sambuco D., Jagsi R. (2014). Women’s participation in the medical profession: Insights from experiences in Japan, Scandinavia, Russia, and Eastern Europe. J. Womens Health (Larchmt).

[6] Bonzini M., Coggon D., Palmer K.T. (2007). Risk of prematurity, low birthweight and pre-eclampsia in relation to working hours and physical activities: A systematic review. Occup. Environ. Med..

[7] Takeuchi M., Rahman M., Ishiguro A., Nomura K. (2014). Long working hours and pregnancy complications: female physicians survey in Japan. BMC Pregnancy Childbirth.

[8] Nomura K., Yamazaki Y., Gruppen L.D., Horie S., Takeuchi M., Illing J. (2015). The difficulty of professional continuation among female doctors in Japan: A qualitative study of alumnae of 13 medical schools in Japan. BMJ Open.

[9] Lerner L.B., Stolzmann K.L., Gulla V.D. (2009). Birth trends and pregnancy complications among women urologists. J. Am. Coll. Surg..

[10] Turner P.L., Lumpkins K., Gabre J., Lin M.J., Liu X., Terrin M. (2012). Pregnancy among women surgeons: Trends over time. Arch. Surg..

[11] Gabbe S., Morgan M.A., Power M.L., Schulkin J., Williams S.B. (2003). Duty hours and pregnancy outcome among residents in obstetrics and gynecology. Obstet. Gynecol..

[12] Finch S.J. (2003). Pregnancy during residency: A literature review. Acad. Med..

[13] Heffner L.J. (2004). Advanced maternal age—How old is too old?. N. Engl. J. Med..

[14] Hsieh T.T., Liou J.D., Hsu J.J., Lo L.M., Chen S.F., Hung T.H. (2010). Advanced maternal age and adverse perinatal outcomes in an Asian population. Eur. J. Obstet. Gynecol. Reprod Biol..

[15] Central Intelligence Agency (2016). The world factbook: birth rate. https://www.cia.gov/library/publications/resources/the-world-factbook/rankorder/2054rank.html.

[16] Department of Household Registration, Ministry of the Interior (2015). Household registration statistics history. http://www.ris.gov.tw/en/web/ris3-english/history.

[17] Lee R., Mason A., members of the NTA Network (2014). Is low fertility really a problem? Population aging, dependency, and consumption. Science.

[18] Nargund G. (2009). Declining birth rate in Developed Countries: A radical policy re-think is required. Facts Views Vis. Obgyn.

[19] Makoto A. (2001). Very low fertility in Japan and value change hypotheses. Rev. Popul. Soc. Policy.

[20] Troske K.R., Voicu A. The effect of children on the level of labor market involvement of married women: What is the role of education?. http://ftp.iza.org/dp4074.pdf.

[21] World Health Organization Demographic and socioeconomic statistics: Fertility rate, 2015, data by World Bank income group. http://apps.who.int/gho/data/view.main.2060.

[22] Chen Y.C., Yeh H.Y., Wu J.C., Haschler I., Chen T.J., Wetter T. (2011). Taiwan’s National Health Insurance Research Database: Administrative health care database as study object in bibliometrics. Scientometrics.

[23] Yang L.Y., Lynn A.M., Chen T.J. (2015). Ambulatory care visits to pediatricians in Taiwan: A nationwide analysis. Int. J. Environ. Res. Public Health.

[24] Sleebos J. (2013). Low Fertility Rates in OECD Countries: Facts and Policy Responses.

[25] Molina Cartes R., González Araya E. (2012). Teenage pregnancy. Endocr. Dev..

[26] McCall S.J., Bhattacharya S., Okpo E., Macfarlane G.J. (2015). Evaluating the social determinants of teenage pregnancy: A temporal analysis using a UK obstetric database from 1950 to 2010. J. Epidemiol Community Health.

[27] Hutchinson A.M., Anderson N.S., Gochnour G.L., Stewart C. (2011). Pregnancy and childbirth during family medicine residency training. Fam. Med..

[28] Willett L.L., Wellons M.F., Hartig J.R., Roenigk L., Panda M., Dearinger A.T., Allison J., Houston T.K. (2010). Do women residents delay childbearing due to perceived career threats?. Acad. Med..

[29] Key T.J., Verkasalo P.K., Banks E. (2001). Epidemiology of breast cancer. Lancet Oncol..

[30] Johnson J.A., Tough S., Society of Obstetricians and Gynaecologists of Canada (2012). Delayed child-bearing. J. Obstet. Gynaecol. Can..

[31] Sutcliffe A.G., Barnes J., Belsky J., Gardiner J., Melhuish E. (2012). The health and development of children born to older mothers in the United Kingdom: Observational study using longitudinal cohort data. BMJ.

[32] Kato T., Yorifuji T., Yamakawa M., Inoue S., Doi H., Eboshida A., Kawachi I. (2017). Association of maternal age with child health: A Japanese longitudinal study. PLoS ONE.

[33] Chang C.F. (2006). The employment discontinuity of married women in Taiwan: Job status, ethnic background and motherhood. Curr. Sociol..

[34] Quansah R., Gissler M., Jaakkola J.J. (2009). Work as a physician and adverse pregnancy outcomes: A Finnish nationwide population-based registry study. Eur. J. Epidemiol..

[35] Henriksen T.B., Hedegaard M., Secher N.J. (1994). The relation between psychosocial job strain, and preterm delivery and low birthweight for gestational age. Int. J. Epidemiol..

